# Relationship Between Neuromodulation and Working Memory in the Prefrontal Cortex: It's Complicated

**DOI:** 10.3389/fncir.2018.00031

**Published:** 2018-04-24

**Authors:** Sarah E. Motley

**Affiliations:** ^1^Department of Neuroscience, Graduate School of Biomedical Sciences and Fishberg, Icahn School of Medicine at Mount Sinai, New York, NY, United States; ^2^California National Primate Research Center, Davis, CA, United States

**Keywords:** working memory, dlPFC, neuromodulation, acetylcholine, dopamine, serotonin, norepinephrine

## Introduction

Determining the neurobiological substrates of working memory is of major clinical significance yet has proven to be a complex undertaking. The dorsolateral prefrontal cortex (dlPFC) is a key site for working memory (Luebke et al., 2010), and the integrity of this cognitive domain has been assessed in both humans and non-human primates using the delayed response (DR) task (Goldman-Rakic, 1990). Critically, this task employs a delay period during which the stimulus is absent and the animal must hold in memory the trial-unique, task-relevant information until the delay ends. Initial investigations showed that acetylcholine (Bartus and Johnson, 1976), dopamine (Arnsten et al., 1994, 1995), and norepinephrine (Arnsten and Goldman-Rakic, 1985) activity influence DR working memory performance. Scientists have examined the dlPFC cellular contributions to working memory and the relationship between neuromodulator receptors and dlPFC cellular activity. Here I synthesize results from across non-human primate studies to examine the complexities of the relationship between neuromodulation and working memory.

## Neural substrates of working memory in the prefrontal cortex

Early studies of working memory in monkeys employed the DR task to examine the dlPFC cellular contributions to this cognitive domain. It was discovered that during the delay period a subset of pyramidal cells in area 46 of the dlPFC is persistently active (Fuster and Alexander, 1971). In spatial versions of the task, delay cells exhibit spatial tuning and fire preferentially for a preferred cue direction. This persistent firing correlates with behavioral performance and is proposed to be the neurological substrate of working memory (Funahashi et al., 1989; Goldman-Rakic, 1996). Computational models predict that persistent firing is dependent upon the NMDA receptor (NMDAR) subunit GluN2B because of its slow kinetics (Wang, 2001), and experimental results confirm this prediction (Wang et al., 2013). Systemic administration of ketamine (NMDA antagonist) decreases behavioral performance and reduces delay cell firing (Wang et al., 2013). Iontophoretic blockade specifically of either receptor subunit GluN2A or GluN2B impairs delay cell firing, with GluN2B blockade also impairing spatial tuning (Wang et al., 2013). Local blockade of the AMPA receptor (AMPAR) has a modest effect on delay cell firing for preferred directions that appears in the latter half of the delay period, suggesting that AMPAR activity is more important for background depolarization (Wang et al., 2013).

In addition to delay cells there are several other cell types in area 46 whose activity supports task performance (Funahashi et al., 1991) fixation cells fire during the duration of the trial and are proposed to play a role in attention (Sun et al., 2017); cue cells fire at the onset of the cue; and, response cells fire during the response portion of the trial. Cue cell firing is decreased by iontophoretic blockade of either GluN2B or AMPAR (Wang et al., 2013). Response cell firing is decreased by iontophoretic blockade of GluN2B, but systemic administration of a general NMDAR antagonist increases both task-related and spontaneous firing of response cells (Wang et al., 2013). Perisaccadic response cells are unaffected by AMPAR blockade, whereas postsaccadic response cells show decreased activity (Wang et al., 2013).

## The role of acetylcholine in working memory delay cell firing

While glutamate drives working memory neurotransmission via AMPAR and NMDAR, dlPFC cellular activity is also heavily influenced by neuromodulators like acetylcholine. The dlPFC receives cholinergic innervation from the nucleus basalis of Meynert in the basal forebrain (Mesulam et al., 1983), and a decrease in the number of nucleus basalis cholinergic cells is correlated with impaired DR average performance (Voytko et al., 1995). Both acetylcholine depletions from the PFC (Croxson et al., 2011) and systemic antagonism of muscarinic receptors (Bartus and Johnson, 1976) cause working memory impairments. Iontophoretic blockade using a general nicotinic receptor antagonist decreases delay cell firing for both preferred and non-preferred directions, and iontophoretic blockade specifically of nicotinic α7 receptor (α7R) reduces delay cell firing only for preferred directions (Yang et al., 2013). Iontophoretic, low-level α7R agonism increases delay cell firing for preferred directions, enhances spatial tuning, and improves behavioral performance whereas high-level agonism impairs behavioral performance (Yang et al., 2013). Local α4β2 receptor (α4β2R) agonism increases delay cell spatial tuning by increasing the firing for preferred directions (Sun et al., 2017). Local α4β2R blockade reverses the effects of α4β2R agonists, and α4β2R agonists restore firing to normal levels after iontophoresis of α4β2R antagonists (Sun et al., 2017). Increasing the cognitive demand by introducing a distractor cue decreases spatial tuning by increasing firing for non-preferred directions and decreasing firing for preferred directions, resulting in impaired behavioral performance. Local α4β2R agonism during the distractor condition restores preferred direction delay cell firing rates to control levels, but has no effect on firing rates for non-preferred directions (Sun et al., 2017). Acetylcholine is known to play a critical role in attention (Sarter et al., 1999), and α4β2R manipulations suggest that acetylcholine is important for working memory function because it supports increased attention needed to maintain persistent firing in the face of a distraction. This idea is further supported by the fact that fixation cell firing, which lasts the duration of the trial and is thought to play a role in attention, is increased by iontophoretic α4β2R agonism for both preferred and non-preferred directions (Sun et al., 2017).

## The role of dopamine in working memory delay cell firing

In addition to cholinergic innervation, the dlPFC receives dopaminergic innervation from the ventral tegmental area (Porrino and Goldman-Rakic, 1982), and systemic agonism of dopamine receptor D1 (D1R) improves DR behavioral performance in both aged monkeys who have naturally occurring low dopamine and in young animals with experimental depletion of catecholamines (Arnsten et al., 1994). Local, low-level D1R agonism increases spatial tuning of delay cells by decreasing firing for non-preferred directions, but high-level agonism decreases spatial tuning by decreasing firing for both preferred and non-preferred directions (Vijayraghavan et al., 2007). Local, low-level D1R antagonism increases firing for preferred directions and modestly decreases firing for non-preferred directions, but high-level antagonism dramatically decreases delay cell firing (Williams and Goldman-Rakic, 1995). Systemic injection of a D1R antagonist impairs behavioral performance (Sawaguchi and Goldman-Rakic, 1991; Arnsten et al., 1994). The inverted-U effects of D1R activity on delay cell activity suggests that dopamine is involved in sculpting spatial tuning such that broad spatial tuning becomes more precise with low-level D1 agonism (Vijayraghavan et al., 2007). Local D2 receptor (D2R) modulation is reported to have no effect on delay cell firing (Wang et al., 2004), but systemic D2R agonism has a biphasic effect on behavioral performance: low doses impair performance but high doses enhance performance (Arnsten et al., 1995), suggesting that D2R activity supports working memory by modulating activity of cell types other than delay cells. Substantial dopamine depletion (90%) produces working memory deficits on the delayed alternation task as severe as surgical ablation of the principal sulcus of the dlPFC (Brozoski et al., 1979).

## The role of norepinephrine in working memory delay cell firing

Similar to D1R agonism, systemic agonism of α2 adrenergic receptor (α2-AR) improves behavioral performance in both aged monkeys who have naturally occurring low norepinephrine and in young animals with experimental depletion of catecholamines (Arnsten and Goldman-Rakic, 1985). The dlPFC receives adrenergic innervation from the locus coeruleus (Porrino and Goldman-Rakic, 1982), and α2-AR agonism increases delay cell firing whereas α2-AR antagonism decreases delay cell firing (Li et al., 1999). Systemic α2-AR agonism at both low and high doses improves working memory performance for aged animals but only high doses improve performance for young animals (Arnsten and Goldman-Rakic, 1985; Franowicz and Arnsten, 1999). Catecholamine depletion impairs working memory, and systemic α2-AR agonism after catecholamine depletion rescues behavioral performance to control levels (Cai et al., 1993). Local α1-AR agonism decreases delay cell firing for preferred directions (Birnbaum et al., 2004), and both local (Birnbaum et al., 2004) and systemic α1-AR agonism impairs working memory (Arnsten and Jentsch, 1997; Mao et al., 1999; Birnbaum et al., 2004). However, behavioral performance is not altered with local blockade of α1-AR (Li and Mei, 1994). Neither systemic (Arnsten and Goldman-Rakic, 1985) nor local injection (Li and Mei, 1994) of a general β-adrenergic antagonist affects behavioral performance. Local blockade of β1-AR enhances working memory (Ramos et al., 2005), and systemic injection of a β2-AR agonist enhances working memory performance in a subset of aged animals (Ramos et al., 2008), suggesting that these two receptors have opposing effects in the dlPFC. Norepinephrine is thought to play a role in working memory function by improving the spatial tuning of delay cell firing thereby decreasing distractibility and improving behavioral performance (Arnsten, 2006). This idea is supported by the effect of α2-AR vs. α1-AR agonism on preferred direction delay cell firing, and is further reinforced by the fact that systemic α2-AR agonism improves behavioral performance when aged animals are tested on the DR task with distractor cues (Arnsten and Contant, 1992). Additionally, depletion of norepinephrine causes modest working memory impairments on the delayed alternation task (Brozoski et al., 1979).

## The role of serotonin in working memory delay cell firing

Compared to the other neuromodulators, significantly less is known about the role of serotonin in working memory. The dlPFC receives serotonergic innervation from the dorsal raphe nucleus (Porrino and Goldman-Rakic, 1982), and local serotonergic 5-HT_2A_ receptor agonism increases delay cell firing for preferred directions and modestly decreases firing for non-preferred directions (Williams et al., 2002). Iontophoretic blockade of 5-HT_2A_ decreases firing for preferred directions and modestly increases firing for non-preferred directions (Williams et al., 2002). General serotonergic stimulation through local, low dose application of either 5-HT or α-methyl-5-HT causes increased firing for preferred directions and a modest increase for non-preferred directions, resulting in overall increased spatial tuning (Williams et al., 2002). However, iontophoretic high dose application of α-methyl-5-HT severely depresses delay cell firing (Williams et al., 2002). It is unclear how serotonin modulates working memory, as a 70% depletion of serotonin in the principal sulcus does not cause working memory impairments on the delayed alternation task (Brozoski et al., 1979). In marmosets, serotonin depletion in the orbitofrontal cortex increases perseverative errors on a serial discrimination reversal task (Clarke et al., 2004, 2007) but does not impair attentional set shifting (Clarke et al., 2005).

## Conclusions: the complicated relationship between neuromodulation and working memory

The complex and varied effects of neuromodulation on dlPFC cellular activity and working memory performance are summarized in Table 1. Although neurotransmitter (AMPA or NMDA) receptor modulation generally affects the firing of area 46 cell types in a similar manner (e.g., antagonism of GluN2B decreases firing for cue cells, delay cells, and response cells Wang et al., 2013), neuromodulator receptor activity has cell type-specific effects. Even more selectively, neuromodulator receptor activity can differently affect subpopulations of the same cell type (e.g., α7R activity affects the firing rate of preferred direction delay cells but has no effect on non-preferred direction delay cells, Yang et al., 2013), and the degree to which neuromodulation affects preferred vs. non-preferred direction delay cells is sensitive to the level of cognitive demand of the task (Sun et al., 2017). Further, a given neuromodulator can be active at a number of receptors, and activation of different receptor subunits can have a dramatically different effect on cell activity (e.g., α1-AR agonism decreases delay cell firing whereas α2-AR agonism increases delay cell firing, Li et al., 1999).

**Table 1:** Effects of neurotransmitter and neuromodulator modulation during a Delayed Response task on cell type-specific firing in the dlPFC and behavioral performance.

**Receptor**	**Drug**	**Effect on cue cell firing rate**	**Effect on preferred direction Delay Cell firing rate**	**Effect on anti-preferred direction Delay Cell firing rate**	**Effect on Response Cell firing rate**	**Effect on Fixation Cell firing rate**	**Effects on Delayed Response performance**	**Citation(s)**
Glutamate GluN2A subunit	Antagonist	?	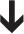	?	?	?	?	Wang et al., 2013
Glutamate GluN2B subunit	Antagonist	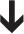	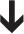	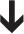	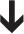	?	?	Wang et al., 2013
Glutamate NMDAR	Antagonist	?	Local: 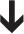 Systemic: 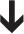	?	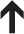	?	Impaired	Wang et al., 2013
Glutamate NMDAR	Agonist	?	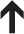	?	?	?	?	Wang et al., 2013
Glutamate AMPAR	Antagonist	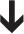	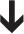 Only in latter half of delay period	No effect	Perisaccadic: no effect; Postsaccadic: 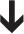	?	?	Wang et al., 2013
Acetylcholine muscarinic receptor	Antagonist	?	?	?	?	?	Impaired	Bartus and Johnson, 1976
Acetylcholine nicotinic receptor	Antagonist	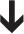	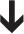	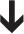	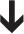	?	?	Yang et al., 2013
Acetylcholine α4β2R	Antagonist	?	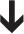	No effect	?	?	?	Sun et al., 2017
“	Agonist	No effect	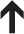	No effect	No effect	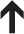	?	Sun et al., 2017
Acetylcholine α7R	Antagonist	No effect	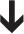	No effect	?	?	?	Yang et al., 2013
“	Agonist	?	Low dose: 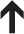 High dose: 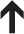	Low dose: No effect High dose: 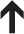	?	?	Low dose: enhanced High dose: impaired	Yang et al., 2013
Dopamine D1R	Antagonist	No effect	Low dose: 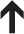 High dose: 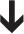	Low dose: No effect High dose: 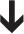	No effect	?	Impaired	Sawaguchi and Goldman-Rakic, 1991; Arnsten et al., 1994; Williams and Goldman-Rakic, 1995
“	Agonist	?	Low dose: No effect High dose: 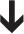	Low dose: 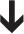 High dose: 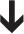	?	?	Enhanced	Arnsten et al., 1994; Vijayraghavan et al., 2007
Dopamine D2R	Antagonist	No effect	No effect	No effect	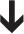 only for preferred direction	?	?	Wang et al., 2004
“	Agonist	No effect	No effect	No effect	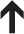 only for preferred direction	?	Low dose: impaired High dose: enhanced	Arnsten et al., 1995; Wang et al., 2004
Norepinephrine α1-AR	Antagonist	?	?	?	?	?	No effect	Li and Mei, 1994
“	Agonist	?	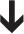	?	?	?	Impaired	Arnsten and Jentsch, 1997; Mao et al., 1999; Birnbaum et al., 2004
Norepinephrine α2-AR	Antagonist	?	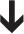	?	?	?	Impaired	Arnsten and Goldman-Rakic, 1985; Li and Mei, 1994; Li et al., 1999; Mao et al., 1999
“	Agonist	?	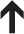	?	?	?	Low dose: no effect High dose: enhanced	Arnsten and Goldman-Rakic, 1985; Cai et al., 1993; Franowicz and Arnsten, 1999; Li et al., 1999; Mao et al., 1999
Norepinephrine general β-AR	Antagonist	?	?	?	?	?	No effect	Arnsten and Goldman-Rakic, 1985; Li and Mei, 1994
Norepinephrine β1-AR	Antagonist	?	?	?	?	?	Enhanced	Ramos et al., 2005
Norepinephrine β2-AR	Agonist	?	?	?	?	?	Enhanced	Arnsten and Goldman-Rakic, 1985
Serotonin 5-HT_2A_R	Antagonist	?	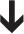	?	?	?	?	Williams et al., 2002
“	Agonist	?	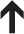	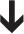	?	?	?	Williams et al., 2002
Serotonergic receptor	Agonist	?	Low dose: 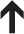 High dose: 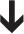	Low dose: 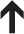 High dose: 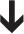	?	?	?	Williams et al., 2002

Although many studies have investigated the role of neuromodulation on delay cell firing, the neuromodulation of other dlPFC cell types remains vastly understudied. Local α4β2 agonism increases fixation cell firing for both preferred and non-preferred directions but has no effect on cue or response cells (Sun et al., 2017). Iontophoretic blockade of α7R has no effect on cue cell firing (Yang et al., 2013). Local D2R modulation affects both perisaccadic and postsaccadic response cell firing but only for preferred directions, with D2R agonism increasing response firing and D2R antagonism decreasing response firing (Wang et al., 2004). Local D1R modulation has no effect on response cell firing, and local D2R modulation has no effect on cue cell firing (Wang et al., 2004). To understand the complex dynamics of the working memory network it is imperative that more work be done investigating the effects of neuromodulation on cue cells, response cells, and fixation cells.

A number of studies use delay cell firing as a proxy for working memory performance. It bears mentioning that there are alternative hypotheses for the significance of persistent delay cell firing other than the maintenance of task-relevant information (Rowe et al., 2000; Tsujimoto and Postle, 2012; Konecky et al., 2017). Nevertheless, studying the effects of neuromodulation on delay cell firing has given insights into potential pharmacological strategies for improving working memory deficits, and the non-human primate model is especially suitable to studying the neurobiological substrates of working memory given the unique qualities of the human and non-human primate dlPFC (Preuss, 1995; Wise, 2017). But local modulation of a receptor is not network-wide modulation and there remains a tremendous knowledge gap in how many of these neuromodulator receptors affect behavioral performance. For therapeutic interventions to be efficacious it is critical that we understand how neuromodulation affects working memory performance especially when systemic administration of a drug is a significantly less invasive therapeutic intervention than local infusion. Building from our current foundation of knowledge, it is important that future studies examine the behavioral effects of neuromodulation as well as the cellular effects on all cell types and not just delay cells.

## Author contributions

The author confirms being the sole contributor of this work and approved it for publication.

### Conflict of interest statement

The author declares that the research was conducted in the absence of any commercial or financial relationships that could be construed as a potential conflict of interest. The handling editor is currently co-hosting a Research Topic with the reviewer SR, and confirms the absence of any other collaboration.
